# Placenta-Mediated Conditions: Past, Present, and Future Perspectives

**DOI:** 10.3390/jcm13164631

**Published:** 2024-08-07

**Authors:** Amihai Rottenstreich

**Affiliations:** 1 Laboratory of Blood and Vascular Biology, Rockefeller University, New York, NY 10065, USA; amichaimd@gmail.com; Tel.: +212-327-7494; Fax: 212-327-7493; 2 Division of Maternal-Fetal Medicine, Department of Obstetrics and Gynecology, Zucker School of Medicine at Hofstra/Northwell, New York, NY, USA

Pregnancy is a highly regulated biological condition in which a successful outcome is heavily dependent on maintaining a delicate balance through maternal–fetal dialog at various levels. Placenta-mediated conditions encompass a variety of complications which account for maternal, fetal, and neonatal morbidity and mortality globally. Up until the latter half of the 20th century, advancements in our understanding of the pathophysiologic mechanisms underlying these disorders were limited. In the last two decades, we have witnessed significant progress in the field as important translational research findings have become increasingly embedded into the care of pregnant patients. These findings have influenced the prevention, diagnosis, and management of different placenta-mediated conditions.

We launched this Special Issue to attract groundbreaking research articles and reviews from across the field. We received many influential articles, five of which were ultimately published.

Diaz Castro et al. evaluated the role of COVID-19 infection in placental energy metabolism and fetal skeletal development. Their important findings support the novel ways in which COVID-19 in affects energy metabolism and bone turnover biomarker homeostasis in the placenta and in colostrum.

Kadivnik and colleagues investigated the association between genetic variants of three cytokine genes encoding for IL-6, IL-10, and TNF-alpha and the occurrence of preterm birth. Their significant findings suggest different opposing roles for two of the studied variants.

Innovatively, Cavanagh et al. explored the utilization of placental shear wave elastography to assess placental function. They could not find any differences in the mechanical properties of placental tissues in pregnancies with and without small-for-gestational-age infants, as evidenced by placental shear wave velocity. The use of elastography as well as other new imaging techniques such as 3D microscopic images for evaluating the placenta will shed light on the mechanisms underlying placenta-mediated conditions.

Placenta-mediated conditions are even more complicated in multifetal pregnancies. Ortiz et al. investigated the role of laser therapy in monochorionic twin gestations with twin–twin transfusion syndrome with coexistent selective fetal growth restriction. Selective fetal growth restriction was found to be an independent negative predictor of donor survival.

The Special Issue also includes a cutting-edge article reviewing the latest scientific literature on the use of low-dose aspirin use preeclampsia prevention. This focused review clarifies the different controversies related to aspirin use including who should be treated, the optimal timing of the initiation and cessation of therapy, and the importance of proper dosing. This focused review provides clinical guidance for challenges that clinicians encounter on a daily basis.

In addition to the many topics discussed in the Special Issues, there are many other advances in the field that are worth mentioning.

Aspirin has shown consistent benefits in the prevention of preterm preeclampsia [1]. Nevertheless, aspirin failure is not uncommon, occurring in up to 30% of high-risk pregnant patients [2]. Moreover, the direct mechanisms through which aspirin exerts its beneficial effects within preeclampsia prevention are still largely unclear. Aspirin was shown to improve the uterine artery Doppler parameters [3], while no effect was shown on the levels of placental biomarkers [4]. This highlights the urgent need for additional modalities to improve the prevention of preeclampsia. The adjunctive role of other agents including low-molecular-weight heparin, calcium, metformin, statins, proton pump inhibitors, L-arginine, immunomodulator, etc., requires further clarification and many ongoing studies are aimed at delineating the role of these agents and developing targeted therapies [5].

Improvements in the diagnosis of preeclampsia were have also been made in the last two decades. Among which the use of placental biomarkers to rule in, rule out, and define the prognosis of preeclampsia have become an integral part of clinical practice [6]. Efforts should be made to ensure that the assessment of these biomarkers is possible across the globe. The use of plasma cell-free RNA signatures to predict the occurrence of preeclampsia is also promising [7].

A precision medicine approach to the management of preeclampsia is evolving. Tailoring antihypertensive treatment based on the maternal hemodynamic profile may potentially improve outcomes [8]. Decisions regarding delivery timing, particularly in cases of preterm preeclampsia, are still a matter of debate. Whether the use of placental biomarkers can help in individualizing care, determining the optimal delivery timing, and improving outcomes remains to be seen [9].

Despite the significant progress made in our understanding of placenta-mediated conditions, the full cellular and molecular mechanisms underlying these disorders, including preeclampsia, and a full insight into the different phenotypes affecting preeclampsia, remain largely undetermined. As pregnancy is believed to be a period which may forecast a woman’s future health, improved prediction, prevention, management, and follow-up may lead to improved long-term outcomes.
